# When Laughter Knocks You Out: A Case of Laughing‐Induced Syncope and a Review of the Literature

**DOI:** 10.1002/ccr3.71297

**Published:** 2025-10-15

**Authors:** Stephanie Abutu, Mohammad El‐Din, Prashanth Raju, Ibrahim Antoun

**Affiliations:** ^1^ Department of Cardiology Kettering General Hospital Kettering UK; ^2^ Department of Cardiovascular Sciences Clinical Science Wing, Glenfield Hospital, University of Leicester Leicester UK

**Keywords:** laughing syncope, loss of consiousness, reflex syncope, situational syncope

## Abstract

Significant implications, particularly in high‐risk settings such as driving. This case highlights the diagnostic challenges in identifying this condition, which necessitate a comprehensive evaluation to rule out structural or electrical cardiac abnormalities and confirm the diagnosis through careful monitoring. The proposed pathophysiology involves exaggerated vagal responses or inappropriate sympathetic withdrawal, although the exact mechanisms remain unclear. Management hinges on a tailored, trigger‐focused approach, emphasizing patient education and behavioral modifications to prevent a recurrence. Increased awareness of laughter‐induced syncope is vital for timely diagnosis, effective management, and the prevention of serious adverse outcomes.

## 1 Introduction

Cardiovascular disease is increasing in prevalence, and its management is challenging, especially in the developing world [1, 2, 3, 4]. Syncope is a transient loss of consciousness due to transient global cerebral hypoperfusion, characterized by rapid onset, short duration, and spontaneous recovery. It is a common clinical problem, accounting for approximately 1% of emergency department visits [5]. The differential diagnosis of syncope is broad and includes reflex (neurally mediated) syncope, orthostatic hypotension, and cardiac arrhythmias, with varying implications for patient prognosis and management [6].

Laughter‐induced syncope, a rare subset of neurally mediated syncope, is an uncommon presentation with limited documentation in the literature [7]. It is considered a subtype of situational syncope, where certain specific triggers, such as coughing, micturition, or swallowing, provoke a vagal response that leads to bradycardia or hypotension. The pathophysiological mechanism involves excessive vagal stimulation or inappropriate sympathetic withdrawal, which leads to transient cerebral hypoperfusion [7].

This case highlights a unique presentation of laughter‐induced syncope in a patient with a history of supraventricular tachycardia (SVT), obesity, and a family history of cardiac disease. It underscores the importance of recognizing rare syncope triggers and tailoring patient counseling to prevent a recurrence. Given this presentation's unusual and potentially hazardous nature, this report aims to contribute to the limited existing data on laughter‐induced syncope and its management, emphasizing the importance of thorough assessment and individualized care.

## 2 Case Presentation and History

The patient is a 57‐year‐old Caucasian female previously managed for SVT. She lives with her daughter and has a body mass index (BMI) of 44.7 kg/m^2^. She does not drink alcohol or smoke cigarettes. Her regular medications include atorvastatin 20 mg once a day (OD), citalopram 10 mg OD, bisoprolol 5 mg OD, and lansoprazole 15 mg OD. Her family history is positive for heart failure in her mother, and her father died of lung cancer in his late sixties. She was referred by her general practitioner (GP) to the urgent cardiology clinic on account of a syncopal episode while driving, leading to a road traffic accident. This episode was witnessed by her daughter, with whom she laughed vigorously when syncope occurred. Fortunately, her daughter was able to steer the car off the road, avoiding a major collision. She, however, sustained a left wrist fracture. She regained consciousness after what her daughter estimated to be approximately 2–3 min. It should be noted that eyewitness accounts may overestimate the duration of transient loss of consciousness; the episode was brief and followed by complete spontaneous recovery without postictal features. She reports having a similar episode in the past, brought on by a laughing spell a while ago at home.

## 3 Differential Diagnosis and Diagnostic Tests

**FIGURE 1:**
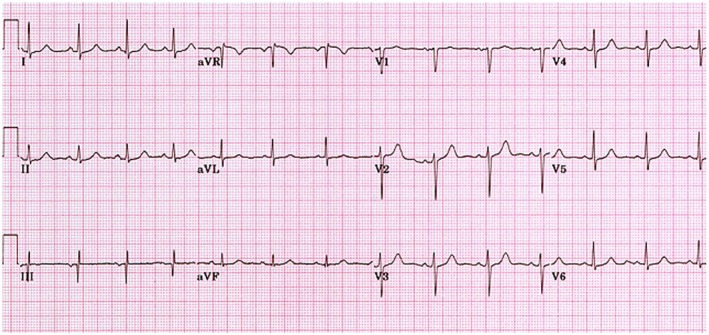
12‐lead electrocardiogram on assessment showing normal sinus rhythm without evidence of arrhythmia or ischemic changes.

Her observations and cardiovascular and neurological examinations were normal. Her resting 12‐lead electrocardiogram (ECG) showed a sinus rhythm without ST changes, QT prolongation, or evidence of pre‐excitation (Figure 1). The echocardiogram showed a structurally normal heart with preserved biventricular systolic function. Her 72‐h heart monitor showed sinus rhythm, including a further episode of syncope induced by laughter. The neurological assessment was unremarkable, with no features suggestive of a seizure, such as tonic–clonic activity, urinary incontinence, tongue biting, or postictal confusion. Her computed tomography of the brain was normal. Routine blood investigations, including a full blood count, renal profile, electrolytes, thyroid function tests, and glucose levels, were all within normal limits, excluding reversible metabolic or endocrine causes of syncope. Given the clear temporal association with laughter and reproduction of syncope on ambulatory monitoring in sinus rhythm, further testing, such as an electroencephalogram, was not pursued.

## 4 Results and Conclusion

The patient was advised against having an extensive laugh, especially outside the house, as this can trigger a blackout. A six‐month follow‐up was reassuring, as the patient experienced no further episodes after avoiding excessive laughter. In accordance with UK Driver and Vehicle Licensing Agency (DVLA) guidance, the patient was advised to refrain from driving until the evaluation was complete and the recurrence risk had been assessed. She was counseled regarding the implications of syncope on driving safety and medico‐legal obligations.

## 5 Discussion

This case highlights a rare and intriguing subset of situational syncope: laughter‐induced syncope. Although syncope is a common clinical presentation, laughter as a trigger is uncommon and scarcely reported in the medical literature [8]. It is also called the Seinfeld syncope after the television show that the patient was watching during the laughter‐induced syncope [9]. This case emphasizes the importance of identifying and understanding unusual triggers to provide appropriate patient management and mitigate risks associated with recurrent episodes.

**FIGURE 2:**
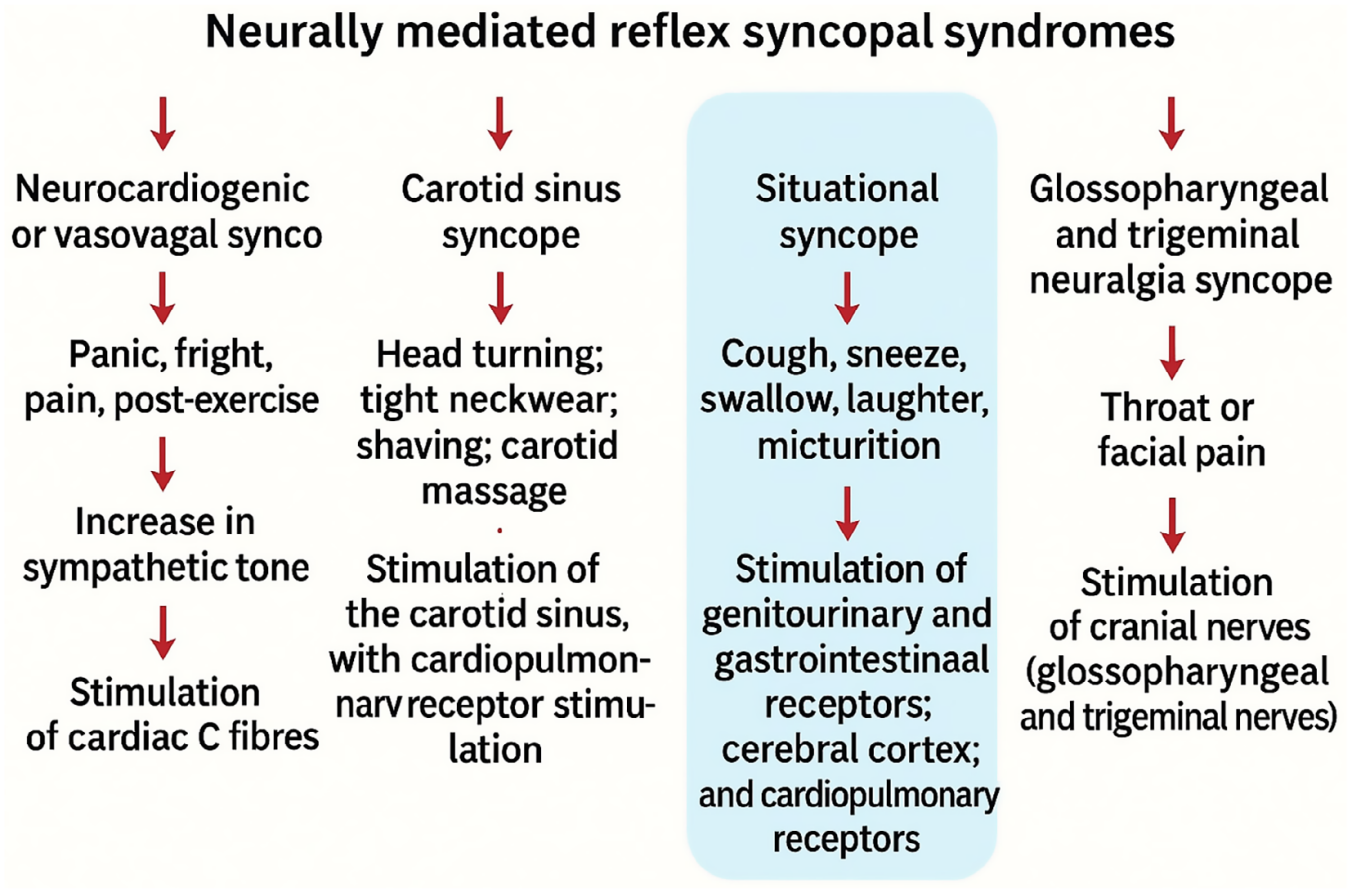
Schematic representation of neurally mediated reflex syncope syndromes. Reflex syncope can be classified into four main forms: Vasovagal, carotid sinus, situational, and cranial nerve‐mediated. Situational syncope is triggered by specific actions such as coughing, sneezing, swallowing, micturition, and, rarely, laughter. Laughter‐induced syncope, as in the present case, is attributed to vagal overactivity or inappropriate sympathetic withdrawal, resulting in transient cerebral hypoperfusion.

Laughter‐induced syncope is a neurally mediated reflex syncope triggered by laughter, likely provoking an exaggerated vagal response or inappropriate sympathetic withdrawal [10]. This leads to bradycardia, vasodilation, or a combination of both, resulting in transient global cerebral hypoperfusion and loss of consciousness (Figure 2). Situational syncope typically involves a precipitating factor—such as coughing, micturition, defecation, or swallowing—that increases intrathoracic pressure, stimulating mechanoreceptors in the thoracic cavity or upper airway [11]. While laughter has been recognized as a similar trigger in rare cases, the exact physiological cascade remains incompletely understood.

This patient's presentation is notable for several reasons. First, laughter‐induced syncope is rare and may be overlooked in initial evaluations. The presence of a previous episode underscores the recurrent nature of this condition if specific triggers are not addressed. Additionally, the association with a road traffic accident highlights the potential danger of this condition if left unmanaged.

The absence of structural cardiac abnormalities or arrhythmic findings on extensive testing, including echocardiography and prolonged cardiac monitoring, supports the diagnosis of situational syncope. Moreover, while unrelated to the episodes, the patient's history of SVT emphasizes the importance of thorough cardiac evaluation to rule out arrhythmic causes.

The management of situational syncope focuses on identifying and avoiding triggers. In this case, the patient was counseled to minimize excessive laughter, particularly in high‐risk situations like driving. Behavioral modifications and trigger avoidance are crucial preventive strategies, as there is no specific pharmacological treatment for situational syncope.

Cardiac monitoring during the symptomatic period confirmed sinus rhythm, effectively excluding arrhythmias as a cause. This reassured the patient and healthcare team, allowing management to focus on education and lifestyle adjustments. Six‐month follow‐up demonstrated the effectiveness of this approach, as the patient experienced no further episodes after adhering to trigger avoidance measures [12]. Comparison with previously reported cases of laughter‐induced syncope provides useful context. Most published cases occurred in middle‐aged men without significant structural heart disease, although instances in women have also been described [13]. Our patient, a 57‐year‐old woman with obesity and a history of SVT, represents a less typical demographic. Recurrence is reported in several cases, often when laughter triggers were not recognized, whereas in our patient, trigger avoidance proved effective during follow‐up. Outcomes in published reports are generally benign when cardiac and neurological causes are excluded, although, as in our case, episodes may lead to secondary harm such as accidents. This underscores the importance of early recognition, counseling, and preventive strategies. The role of obesity in this case warrants consideration, as it has also been described in previous case reports [14]. It may predispose individuals to obstructive sleep apnoea or altered autonomic regulation, potentially exacerbating syncope risk [15].

Furthermore, the patient's positive family history of heart failure necessitates continued surveillance for long‐term cardiovascular risk, even in the absence of immediate structural abnormalities. This case highlights the importance of considering rare triggers, such as laughter, in the differential diagnosis of syncope. The contributory role of comorbidities in this case also warrants consideration. A comprehensive evaluation, including a detailed history, cardiac and neurological assessments, and targeted investigations, is essential for an accurate diagnosis and effective management. Obesity is a recognized risk factor for obstructive sleep apnea, which is associated with autonomic dysregulation and may predispose to syncopal episodes. Although not formally assessed, undiagnosed sleep apnea could have compounded susceptibility [16]. The patient's history of SVT, although not directly related to her syncope, highlights the importance of thorough arrhythmic evaluation to exclude overlapping cardiac causes. In addition, medications such as bisoprolol may blunt compensatory sympathetic responses, while selective serotonin reuptake inhibitors such as citalopram have been linked to QT prolongation and altered autonomic tone. Although these were not implicated in the present case, they may contribute to the overall autonomic milieu and should be considered in the clinical assessment of syncope. Through patient education and lifestyle modifications, recurrent episodes can often be prevented, improving patient safety and quality of life. While autonomic testing, such as heart rate variability analysis or head‐up tilt testing, was not performed in this case, these modalities could provide further insights into the autonomic mechanisms underlying laughter‐induced syncope. Their inclusion in future assessments may help delineate the relative contributions of vagal activation and sympathetic withdrawal, potentially advancing our understanding of this rare condition. Although seizure was considered in the differential diagnosis, the absence of convulsive features, tongue biting, or postictal state, together with cardiac monitoring during a symptomatic episode, supported the diagnosis of syncope. Although the daughter estimated the duration of unconsciousness at 2–3 min, this may represent an overestimation, as is common in witness accounts.

Typical syncope episodes last less than 1–2 min, and the absence of postictal features supports syncope rather than seizure as the underlying diagnosis. However, an electroencephalogram was not performed in this case, which may be viewed as a limitation. It is important to differentiate laughter‐induced syncope from gelastic seizures, a rare form of epilepsy characterized by inappropriate or unprovoked episodes of laughter, typically arising from hypothalamic hamartomas or other cortical lesions. Automatisms, impaired awareness, or postictal states often accompany gelastic seizures [17].

In contrast, laughter‐induced syncope occurs exclusively during episodes of voluntary laughter, resulting in a transient loss of consciousness due to autonomic mechanisms. In our case, the clear temporal association with laughter, absence of automatisms or postictal confusion, and confirmation of sinus rhythm during a reproduced episode support syncope rather than seizure as the underlying diagnosis. Such investigations may be appropriate in cases with diagnostic uncertainty to conclusively exclude seizure disorders. Future research should aim to elucidate the precise mechanisms of laughter‐induced syncope and explore potential therapeutic strategies.

## Author Contributions

**Stephanie Abutu:** conceptualization, data curation, writing – review and editing. **Mohammad El‐Din:** writing – review and editing. **Prashanth Raju:** writing – review and editing. **Ibrahim Antoun:** supervision, writing – original draft.

## Consent

The patient gave written informed consent to publish this report in accordance with the journal's patient consent policy.

## Conflicts of Interest

The authors declare no conflicts of interest.

## Data Availability

Data relating to this study is available upon reasonable request from the corresponding author.
